# Ataxia as the Major Manifestation of Fragile X-Associated Tremor/Ataxia Syndrome (FXTAS): Case Series

**DOI:** 10.3390/biomedicines8050136

**Published:** 2020-05-25

**Authors:** Maria Jimena Salcedo-Arellano, Ana Maria Cabal-Herrera, Nattaporn Tassanakijpanich, Yingratana A. McLennan, Randi J. Hagerman

**Affiliations:** 1 Department of Pediatrics, University of California Davis School of Medicine, Sacramento, CA 95817, USA; mjsalcedo@ucdavis.edu (M.J.S.-A.); yamclennan@ucdavis.edu (Y.A.M.); 2 MIND Institute, University of California Davis, Sacramento, CA 95817, USA; ana.cabal@correounivalle.edu.co (A.M.C.-H.); tnattaporn.ped@gmail.com (N.T.); 3 Group on Congenital Malformations and Dysmorphology (MACOS), Faculty of Health, Universidad del Valle, Cali, Valle del Cauca 760041, Colombia; 4 Department of Pediatrics, Faculty of Medicine, Prince of Songkla University, Songkhla 90110, Thailand

**Keywords:** fragile X-associated tremor and ataxia syndrome, *FMR1*, ataxia, neurodegeneration

## Abstract

Fragile X-associated tremor and ataxia syndrome (FXTAS) is a neurodegenerative disease developed by carriers of a premutation in the fragile X mental retardation 1 (*FMR1)* gene. The core clinical symptoms usually manifest in the early 60s, typically beginning with intention tremor followed by cerebellar ataxia. Ataxia can be the only symptom in approximately 20% of the patients. FXTAS has a slow progression, and patients usually experience advanced deterioration 15 to 25 years after the initial diagnosis. Common findings in brain imaging include substantial brain atrophy and white matter disease (WMD). We report three cases with an atypical clinical presentation, all presenting with gait problems as their initial manifestation and with ataxia as the dominant symptom without significant tremor, as well as a faster than usual clinical progression. Magnetic resonance imaging (MRI) was remarkable for severe brain atrophy, ventriculomegaly, thinning of the corpus callosum, and periventricular WMD. Two cases were diagnosed with definite FXTAS on the basis of clinical and radiological findings, with one individual also developing moderate dementia. Factors such as environmental exposure and general anesthesia could have contributed to their clinical deterioration. FXTAS should be considered in the differential diagnosis of patients presenting with ataxia, even in the absence of tremor, and *FMR1* DNA testing should be sought in those with a family history of fragile X syndrome or premutation disorders.

## 1. Introduction

The fragile X-associated tremor and ataxia syndrome (FXTAS) is a neurodegenerative disease developed by fragile X mental retardation 1 (*FMR1)* premutation carriers [1,2]. The premutation status is defined by the CGG trinucleotide expansion in the untranslated region of the *FMR1* gene located in the X chromosome ranging from 55 to 200 repeats and characterized by the absence of hypermethylation within the promoter region [3]. Expansions greater than 200 CGG repeats are the cause of fragile X syndrome (FXS). The neurodegeneration found in FXTAS is associated with the toxic effects induced by a two- to fourfold increase in the production of messenger RNA (mRNA) [4]. The onset of symptoms of FXTAS often occurs during the seventh decade of life in 40% of male and 16% of female *FMR1* premutation carriers [5]. The classic presentation of neurological symptoms begins with a subtle intention tremor that might be only perceived by the patient while writing or holding a cup, followed by an ataxic gait often referred by the patient or the caregiver as lack of balance and/or frequent falls. Other common associated symptoms are parkinsonism, cognitive decline, mood disorders and peripheral neuropathy [6]. FXTAS has a slow progression and patients experience advanced deterioration 15 to 25 years after initial diagnosis. The clinical presentation is very heterogeneous but the syndrome rarely presents with one dominant symptom in the absence of others. In this case series, we present three patients with an atypical clinical presentation of mild to severe ataxia without an objectively measured tremor.

## 2. Materials and Methods

The patients included in this report were active research participants in a longitudinal genotype phenotype study for older carriers of the *FMR1* premutation with neurological problems who signed an Institutional Review Board (IRB)-approved informed consent (IRB 254134 last approved on 5/19/2020) to have their medical histories reviewed and their cases published for medical and scientific purposes. The brain images presented were also part of the research protocol; images were captured using a 3T Siemens TIM Trio MRI System available at the Imaging Research Center—University of California Davis Health.

Assessments included the Mini-Mental State Exam (MMSE)—the maximum MMSE score is 30 points. A score of 20 to 24 suggests mild dementia, 13 to 20 suggests moderate dementia, and less than 12 indicates severe dementia [7]; the Wechsler Adult Intelligence Scale fourth edition (WAIS-IV) Full Scale IQ (FSIQ)—a score less than 70 indicates intellectual disability, 70 to 79 defines borderline, 80 to 119 equals average IQ, and a score greater than 119 designates superior IQ [8]; and the Behavioral Dyscontrol Scale (BDS-2)—a nine-item assessment used to measure executive functioning deficits, wherein in predicting functioning in daily life, all items are scored from 0 to 3, for a maximum total score of 27 [9]. All three cases also had a licensed psychologist evaluate any psychiatric diagnoses via the Structured Clinical Interview for DSM-5 (SCID-5-RV) [10].

Motor symptoms were evaluated by using Kinesia^TM^ One (Great Lakes NeuroTechnologies, Software Version: 2.0.0), a web-based application that objectively measures tremor through an accelerometer worn on a participant’s finger while they are assessed for Parkinson’s-like symptoms. The medical and neurological examination were conducted by a physician experienced with FXTAS (RJH).

FXTAS was staged (range I–VI), according to functional impairment, where (I) represents a subtle or questionable gait instability and/or tremor, (II) represents a minor but clearly detectable balance problem and/or tremor with minimal interference in daily activities, (III) denotes moderate ataxia and/or tremor that significantly interferes with daily activities and reporting occasional falls, (IV) signifies severe ataxia and/or tremor that requires the use of a mobility aid (walker or cane), (V) denotes a daily use of a wheelchair for mobilization, and (VI) denotes the individual is bedridden [11].

## 3. Case Presentation

### 3.1. Case 1

This participant is a 67-year-old retired male who has the fragile X premutation with 126 CGG repeats. He has a history of idiopathic skeletal hyperostosis involving his back, causing intermittent pain and more often stiffness. He is also diagnosed with sleep apnea and he has been on continuous positive airway pressure (CPAP) for the last 3 years. His neurological symptoms began with mild gait problems at the age of 57. His wife noticed these gait problems initially and he has had three significant falls in the last few years. He does not use a cane. He denies swallowing problems, but he states that his memory has been spottier in the last 2 or 3 years. He had the onset of erectile dysfunction at age 57. Since retirement, he does not enjoy cooking and gardening, which he enjoyed in the past. He tires easily and takes 2 h naps throughout the day.

His present medication includes atorvastatin (40 mg a day), Centrum 50, krill oil, and an antihistamine at bedtime when needed for seasonal allergies. He drinks 2–4 beers per day. There is no history of seizures, cardiac problems, hypertension, cancer, diabetes, or autoimmune problems. He receives physical therapy twice a week to help with his balance difficulties. Family history includes his wife and three daughters who are obligate carriers and one unaffected son. His brother’s granddaughter has fragile X syndrome and she was the identified proband in this family.

On examination, vital signs were within normal limits and his body mass index (BMI) was 30. Deep tendon reflexes were hypo-reflexic in the upper extremities and the left knee, and absent at the ankles. He had decreased vibration sense in the right leg and pinprick sensation was decreased to about 25% of normal level in the lower extremities. He did not demonstrate dysdiadochokinesis, and finger-to-nose was normal without a tremor seen. He had difficulty with tandem walking and was only able to take four steps and was unstable. His heel-to-shin movements demonstrated a mild ataxia bilaterally. He had a positive snout reflex for greater than four taps, and absent jaw jerk and a positive palmomental reflex on the right but not on the left. The rest of the exam was unremarkable (additional information is found in Table 1).

**Figure 1:**
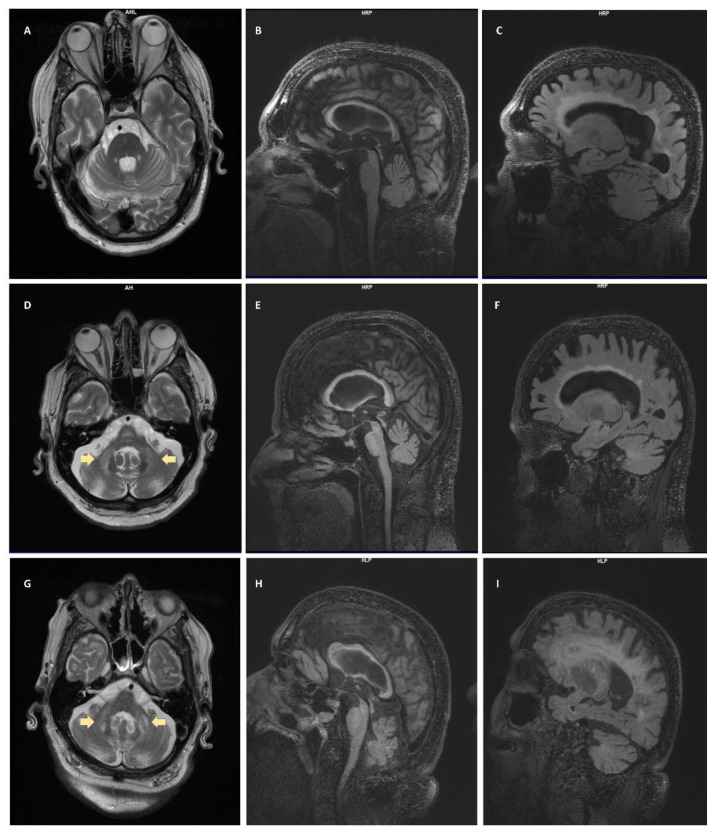
Radiological Evaluation. **Case 1** (**A**) T2- weighted turbo spin echo (T2-TSE) no middle cerebellar peduncle (MCP) sign; (**B**) T2-weighted-Fluid-Attenuated Inversion Recovery (T2-FLAIR) splenium and genu signs; (**C**) T2-FLAIR general atrophy, ventriculomegaly. **Case 2** (**D**) T2-TSE MCP sign (arrows); (**E**) T2-FLAIR splenium and genu signs; (**F**) T2-FLAIR general atrophy, ventriculomegaly, periventricular white matter disease (WMD). **Case 3** (**G**) T2-TSE MCP sign (arrows); (**H**) T2-FLAIR splenium and genu signs; (**I**) T2-FLAIR general atrophy, ventriculomegaly, periventricular WMD.

MRI showed significantly dilated ventricles with a moderate degree of white matter disease in the periventricular area that extended frontally. There was involvement of the splenium, and the corpus callosum was thin for his age; there was also a minimal involvement of the insula with white matter disease and increased perivascular spaces, particularly in the basal ganglia. In addition, suggestive iron deposition was observed in the putamen. There was a mild to moderate degree of atrophy in the cerebrum and increased white matter disease in the deep cerebral white matter. He did not have the middle cerebellar peduncle (MCP) sign (see Figure 1A–C).

On cognitive testing using the WAIS IV, full-scale IQ was 107. BDS-2 was 20 out of 27, suggesting some executive function deficits, whereas MMSE was 29 out of 30. Upon completion of the medical evaluation, he met criteria for probable stage III FXTAS and he also met criteria on the SCID-5 for minor depression (see Table 2 for additional information).

### 3.2. Case 2

This participant is a 68-year-old retired male with a diagnosis of FXTAS (94 CGG repeats) in addition to mild cognitive impairment. His medical history includes a normal childhood and he was in good health until his 50s when he was diagnosed with gastroesophageal reflux disease, hypertension, and type 2 diabetes mellitus. His only health related complaint from early adulthood was recurrent headaches presenting multiple times per week that were associated with sensitivity to light. At age 60, he underwent prostatectomy to treat prostate cancer. He was put under prolonged general anesthesia due to complications during the procedure. After this surgery, he experienced a faster progression of neurological symptoms and he became intolerant to temperature changes. At age 63, he developed a shuffling gait and balance problems, and this led to the use of a cane at age 65 and then a walker at age 66; due to frequent falls he had been using a wheelchair for the past 6 months. He had cancer recurrence at age 65 and was treated with radiation therapy in the prostate area. Subsequently, he developed bladder incontinence and on occasion bowel incontinence. He also reported an onset of handwriting problems at age 65, and a very minor tremor at age 67. He has experienced tinnitus since age 50 and hearing loss in the last 5 years. Swallowing difficulties and choking on food started at age 66, leading to an episode of aspiration pneumonia.

His current daily medications include omeprazole (20 mg), aspirin (81 mg), lisinopril (40 mg), amlodipine (5 mg), metformin ER extended-release (1000 mg), and glimepiride (20 mg). He takes acetaminophen for recurrent headaches and chronic back pain. He was prescribed a 2-month trial of carbidopa/levodopa for his parkinsonian symptoms without beneficial effects. He does not drink alcohol. He denies cardiac and immunological problems. He is married and has an unaffected son, who has been diagnosed with attention deficit disorder (ADD), and a carrier daughter that has experienced health problems related to the premutation, including fragile X-associated primary ovarian failure (FXPOI) and fragile X-associated neuropsychiatric disorders (FXAND). Family history is also remarkable in terms of a brother who died of multiorgan failure in his 70s.

On examination, vital signs were normal, with a controlled blood pressure and appropriate weight; calculated BMI was 26. Neurological evaluation demonstrated decreased smell sensation to rubber (but he can smell coffee), slow eye movements, poor pupil reactivity to light, and hypoactive gag reflex. Strength was found to be mildly decreased in upper and lower extremities, with the presence of paratonia in the upper extremities. Deep tendon reflexes were hypoactive at the knees and absent at the ankles; he had a positive Babinski reflex on the left and he experienced significant jerking of his feet with the Babinski test. Temperature sensation was normal, and there was a slight decrease in vibration sensation in the feet and approximately 30% abnormal scores for both position and pinprick sensation bilaterally in the feet. He demonstrated significant dysdiadochokinesis, which was worse in his nondominant (right) hand; and finger-to-nose testing revealed a very minimal tremor also affecting the nondominant hand; he was unable to walk independently due to weakness and instability. There was very minimal postural tremor and no rest tremor, although he did have parkinsonian features including rigidity, bradykinesia, bradypsychia, and masked faces. Primitive reflexes demonstrated a positive snout reflex for greater than four taps and a positive palmomental reflex on the left; both hands had significant jerks with stroking.

Brain imaging demonstrated a significant MCP sign with white matter disease throughout the pons; there was also severe insular involvement and a thin corpus callosum, and both the splenium and genu demonstrated white matter disease. Cerebral ventricles were considerably enlarged with periventricular white matter disease, and prominent perivascular spaces were seen throughout the cerebrum (see Figure 1D–F).

His WAIS IV full-scale IQ score was 90; BDS-2 was 15 out of 27, demonstrating significant executive function deficits; MMSE was 27 out of 30 (see Table 2 for additional information). In summary, this participant has definite stage V FXTAS with significant involvement of the CNS, including dilated ventricles and widespread white matter disease. He was found to have severe ataxia, mild cognitive impairment, peripheral neuropathy, and parkinsonian features upon examination.

### 3.3. Case 3

The last case is a 74-year-old retired premutation male carrier who is diagnosed with FXTAS and has 91 CGG repeats. This individual has a history of significant ataxia, which began when he was 62 years of age. Initially he started using a cane consistently (67 years old) and then began using a walker (70 years old) before progressing to a wheelchair (73 years old). Over the past 5 years he has suffered several falls that have necessitated surgery, including a Le Fort fracture of the skull, a fractured wrist, a fractured pelvis, and a fractured ankle. He mentioned that he has had some neuropathy symptoms such as pain in his fingertips, but this was after fracturing his wrist. He also has some partial back pain but denies any overall chronic pain. Towards the end of the day, he complains of dizzy spells and becomes tired and subsequently is more prone to falling.

He has a history of kidney stones and nasal polyps, which were removed. His cardiovascular history is significant and includes a myocardial infarction at age 48 after 30 years of smoking. After he underwent a quadruple bypass heart surgery, he stopped smoking and began to treat his high blood pressure with antihypertensives (Terazosin 10 mg in the morning, then alternating with 5 mg every other day). Other medications include simvastatin for hypercholesterolemia and a combined fluticasone furoate and vilanterol inhaler for maintenance of chronic obstructive pulmonary disease. At age 64, he started experiencing memory deficits and complained of anxiety, moodiness, and irritability. These symptoms were managed with memantine and venlafaxine. However, he experienced moderate side effects, including dizziness and more problems with his balance, resulting in cessation of the treatment within a year.

On examination, he was found to have a controlled blood pressure and a normal heart rate. His BMI was found to be 33. Neurological examination revealed anisocoria, and right pupil is slightly bigger and hyporeactive. He also had minimal lateral gaze nystagmus. His hearing on the left side was normal but was decreased 50% on the right. His gag was intact, and his tongue had normal strength, but his articulation was problematic. Muscle strength was normal in the upper extremities. During alternating movements, he had significant increased tone on the right side when drawing a circle with the left hand. Deep tendon reflexes were hypoactive in the upper extremities, and at the knees, the ankle reflexes were absent bilaterally. Temperature sensation was found to be absent in the lower extremities, vibration sense was diminished in the left foot, and pinprick sensation was also reduced bilaterally in the feet. He demonstrated significant dysdiadochokinesis bilaterally. Pull test was positive, and was is unstable while standing. No observable tremor was detected; however, he had significant finger-to-nose dysmetria bilaterally. Assessment of primitive reflexes was inadequate because tapping to the upper jaw with closed lips elicited pain, but the snout reflex appeared to be positive.

Cognition has declined gradually, specifically short-term memory. Verbal Comprehension Index (VCI) on WAIS-IV decreased from 107 to 89 and working memory decreased from 102 to 74 in the last 10 years (see Table 2 for additional information). He scored 18/30 on the MMSE and he was diagnosed with moderate dementia.

Magnetic resonance imaging of the head showed confluence of the cortical white matter disease that was seen 10 years ago, with involvement of the insula and severe involvement in the splenium and genu, and there was also an MCP sign. He had significant cortical atrophy, consistent with the cognitive decline his family reported, as well as mild cognitive impairment scores (see Figure 1G–I).

## 4. Discussion

The three presented patients have common features of FXTAS, which include motor symptoms of ataxia, cognitive decline, peripheral neuropathy, parkinsonism, and autonomic dysfunction. Interestingly, ataxia is the predominant motor problem in our cases, which is distinct from the typical presentation of FXTAS in which tremor usually precedes or co-occurs with ataxia [12,13]. In a systematic review of literature conducted by Zhao and colleagues, they found that 85.2% of patients with FXTAS reported tremor and 74.6% of them had a family history consistent with FXS or premutation disorders, which highlights that most patients will complain of tremors but a minor percentage will have another presenting symptom with no concurring tremor [14]. Case 1 had an early onset of neurological symptoms and unusual radiological findings—no MCP sign, mild cerebral atrophy, and signs suggestive of accumulation of iron in the basal ganglia. In case 2, the worsening of symptoms seemed to be triggered by general anesthesia, and he presented with a constellation of neurological symptoms including hearing loss, impaired taste, and recurrent headaches. In addition, he has experienced a fast progression of the disease, requiring the use of a walker only 3 years after the onset of ataxia. In case 3, the initial clinical presentation consisted of the onset of ataxia accompanied by mood disturbances and cognitive decline. After 12 years of substantial progression of the disease, he has not developed tremor; however, his cognitive dysfunction has advanced to moderate dementia.

### 4.1. Ataxia in FXTAS

Chronic progressive cerebellar ataxia is a core symptom in FXTAS, and ataxia could be the only symptom in approximately 20% of patients [2]. A cross-sectional study [6] found that 55% of the premutation males age ≥ 50 years had gait instability in contrast to only 17% of age-matched control males. Moreover, the premutation males were 13 times more likely to have falls compared with controls. Penetrance of ataxia in the male carriers was found to increase overtime. The frequency of gait instability according to self-reported symptoms was 17% in those aged 50–59 years, steadily increasing to 100% in those over 80 years old [6]. On the other hand, approximately 1% to 2% of individuals presenting with ataxia have a positive *FMR1* DNA testing result [15].

The average age of onset of ataxia in FXTAS is 63.6 ± 7.3 years [12,16]. FXTAS manifestations and radiographic findings may also mimic multiple system atrophy (MSA) cerebellar subtype, although FXTAS tends to progress slower than typical MSA [17]. *FMR1* DNA testing is recommended in individuals aged ≥ 50 years old with either unexplained cerebellar ataxia or atypical MSA cerebellar subtype [15,17]. The presence of tremor and neuropathy as well as family history of FXS should heighten consideration of FXTAS in the differential diagnosis of ataxia.

Moreover, decreased deep tendon reflexes and peripheral sensation on examination indicate peripheral neuropathy, which interferes with the postural stability of patients with FXTAS [18]. Cognitive function, which has been reported to be impaired once ataxia is detected [13], also compromises mobility [19]. Another important feature in FXTAS is a deficit in executive function [20], which is demonstrated in our three cases by their low BDS-2 scores. It has even been suggested that executive dysfunction is the primary cognitive deficit in patients with FXTAS and that it mediates the other cognitive deficits [21].

### 4.2. Evaluation of Ataxia and Contributing Factors for a Faster Progresion in FXTAS

Difficulty in tandem walking, increased gait variability, decreased gait speed, and a lengthened time for movement transition can be the initial and subtle signs of ataxia [13,22]. Dysdiadochokinesia and unstable movement on heel-to-knee-to-shin testing can also be present [13]. Our cases had a more rapid neurological progression than typical individuals with FXTAS. A retrospective cohort study in 55 FXTAS males described the natural history of FXTAS [12]. Falls presented on average 6 years after initial motor problems began, and walking aid was required 15 years after the onset.

Predictive factors for FXTAS have been sought. CGG repeat length positively correlates with earlier onset of FXTAS [16], particularly in a CGG expansion > 70 repeats [23]. The relationship between increased CGG repeats and severity of ataxia has also been observed [24]. In addition, more CGG repeats are associated with reduced cerebellar volume, which is linked to increased postural sway in the premutation males [25]. mRNA was hypothesized to be a predictor of clinical severity, and elevated mRNA level supports the RNA toxicity mechanism in FXTAS. However, mRNA level is variable in different tissues or even in each brain region, and therefore leukocyte mRNA level is unable to represent the mRNA levels in brain tissue [24].

Precipitating factors of FXTAS progression were discerned in our cases. Hypertension, diabetes, hyperhomocysteinemia, and sleep apnea can accelerate white matter change in the brain [26,27,28,29]. General anesthesia, particularly isoflurane and sevoflurane, may interfere with neuronal calcium regulation and mitochondrial function in the premutation carriers and can lead to postoperative cognitive dysfunction [30]. Moreover, chronic alcohol intake and cigarette smoking also hasten progression of FXTAS [31]. These problems are not uncommon in individuals with the premutation; therefore, addressing and managing these problems is fundamental to delay the progression of FXTAS.

### 4.3. Neuroradiologic Findings

The first radiological findings described in FXTAS were seen in males; bilateral hyperintensities of the middle cerebellar peduncles (known as MCP sign) on T2-weighted magnetic resonance or FLAIR images were defined as the hallmark of FXTAS and were included as major radiological criteria for diagnosis [2,32]. Cases 2 and 3 presented with the MCP sign, and therefore this finding categorized them with a definite FXTAS diagnosis. Although it is considered as a hallmark of FXTAS, the MCP sign is only found in 60% of males [5,33] and it is uncommon in females, with approximately 13% displaying the MCP sign [34,35]. It can rarely be present in asymptomatic carriers [33], and thus it must be interpreted along with clinical manifestations (e.g., cerebellar ataxia, scanning speech, vertigo). White matter lesions are also commonly observed; the presence of such in the splenium of the corpus callosum and in cerebral white matter constitute minor neuroradiological criteria for FXTAS [32]. They are commonly reported in the periventricular white matter, as seen in our three cases, as well as in the brainstem. White matter lesions in the splenium of the corpus callosum have been identified as useful signs to aid in the diagnosis of FXTAS, and previous studies have demonstrated a high prevalence in patients with FXTAS [36]. We were able to identify this sign in our three cases (see Figure 1). It has been suggested that neuroradiological abnormalities in the genu of the corpus callosum and in the MCP may play an important role in cognitive impairment [37].

Several studies in patients with FXTAS have found cortical atrophy, especially in the dorsomedial frontal and parietal regions, the insula, and medial temporal regions [38,39]. Volume loss in the thalamus and the striatum has also been reported to be increased compared with premutation carriers without FXTAS [38]. Volume measurements of thalamus, putamen, and caudate have been found to have a negative correlation with FXTAS stage [38]. Brain atrophy was observed in all the cases included in this series. Cerebellar atrophy was seen as well, and it has been associated with postural instability [25]. We additionally identified ventricular enlargement and thinning of the corpus callosum, which have been reported previously in patients with FXTAS [40,41].

Iron dysregulation is a key component of FXTAS pathophysiology [42]; a study conducted in post-mortem FXTAS specimens found increased iron deposition in the putamen of FXTAS cases [43]. Iron deposition and atrophy of the putamen has also been shown in MRI of patients with FXTAS [38], as revealed in case 1 during radiological evaluation.

These radiological abnormalities are important in the diagnosis of FXTAS, correlating with clinical manifestations such as motor symptoms [44], postural instability [25], and deficits in cognitive and executive functions [39,45].

## 5. Conclusions

This case series details the research findings of three male premutation carriers with FXTAS showing an atypical clinical profile. FXTAS most commonly presents with kinetic tremor, which is followed a few years later by the onset of ataxia. Here, we highlighted the importance of suspecting FXTAS in patients presenting with gait deficits or ataxia as the initial symptom of neurodegeneration, as up to 20% of patients with FXTAS can develop ataxia without concomitant tremor [2]. It is equally important to suspect FXTAS when the patient presents with cognitive decline and gait abnormalities because approximately 40% of patients with FXTAS develop dementia in advanced stages of the disease [20]. Exposure to toxins, chemotherapy, chemical agents, chronic substance use, and general anesthesia have been associated with disease progression, as exemplified in one of our cases. Clinicians must be aware of the accompanying signs and symptoms of ataxia such as cognitive decline, deficits in executive function, and dysautonomia. Neuroradiological findings most commonly include white mater lesions and brain atrophy. Although the presence of the MCP sign constitutes a major radiological criterion for FXTAS, it is only present in 60% of males and it can rarely be found in asymptomatic carriers. FXTAS should be in the differential diagnosis of patients presenting with ataxia, especially if there is a family history of FXS or associated premutation disorders.

## Figures and Tables

**Table 1:** Summary of case information.

Case	CGG Repeat Size	Age of Onset	Presenting Neurologic Symptom	Additional Medical Conditions	Labs	MRI Findings	Current FXTAS Stage
1	126	57	Gait problems	Skeletal hyperostosis, sleep apnea, erectile dysfunction, minor depression	CBC: normal CMP: normal Homocysteine: 9.2 TSH: normal HbA1C: 5.9%	Ventriculomegaly, WMD, splenium sign, mild–moderate cerebral atrophy	III
2	94	63	Gait and balance problems	GERD, hypertension, type 2 DM, hearing loss, swallowing problems	CBC: normal CMP: normal Homocysteine: 14.3 (high) TSH: normal HbA1C: 6.2%	MCP sign, WMD, splenium and genu sign, thinning of corpus callosum ventriculomegaly, prominent perivascular spaces	V
3	91	62	Ataxia	CAD, hypertension, hypercholesterolemia, anxiety, irritability, moderate dementia	CBC: normal CMP: normal Homocysteine: 10.7 TSH: normal HbA1C: 6.3%	WMD, splenium sign, cortical atrophy, MCP sign	V

Note: GERD: gastroesophageal reflux disease; DM: diabetes mellitus; CAD: coronary artery disease; CBC: complete blood count; CMP: comprehensive metabolic panel; TSH: thyroid stimulating hormone; MCP: middle cerebellar peduncles, WMD: white matter disease.

**Table 2:** Summary of cognitive and motor scores.

Case	BDS-2	WAIS-IV FSIQ	MMSE	Handedness	Kinesia Rest Tremor	Kinesia Postural Tremor	Kinesia Kinetic Tremor
1	20/27	107	29/30	Right	RH: 0.06 LH: 0.13	RH: 0.00 LH: 0.00	RH: 1.89 LH: 1.89
2	15/27	90	27/30	Right	RH: 0.24 LH: 0.23	RH: 0.22 LH: 0.38	RH: 1.58 LH: 1.10
3	4/27	67	18/30	Left	RH: 0.19 LH: 0.00	RH: 0.17 LH: 0.04	RH: 1.28 LH: 1.17

Note: Kinesia-One is a wearable device used to objectively measure motor tasks related to Parkinson’s disease. It is based on an algorithm that scores movement on a scale of 1–4. Score > 2 is considered clinical. RH: right hand; LH: left hand.

## References

[1] Hagerman R.J., Leehey M., Heinrichs W., Tassone F., Wilson R., Hills J., Grigsby J., Gage B., Hagerman P.J. (2001). Intention tremor, parkinsonism, and generalized brain atrophy in male carriers of fragile X. Neurology.

[2] Jacquemont S., Hagerman R.J., Leehey M., Grigsby J., Zhang L., Brunberg J.A., Greco C., Des Portes V., Jardini T., Levine R. (2003). Fragile X premutation tremor/ataxia syndrome: Molecular, clinical, and neuroimaging correlates. Am. J. Hum. Genet..

[3] Reiss A.L., Freund L.S., Baumgardner T.L., Abrams M.T., Denckla M.B. (1995). Contribution of the FMR1 gene mutation to human intellectual dysfunction. Nat. Genet..

[4] Tassone F., Hagerman R.J., Taylor A.K., Gane L.W., Godfrey T.E., Hagerman P.J. (2000). Elevated levels of FMR1 mRNA in carrier males: A new mechanism of involvement in the fragile-X syndrome. Am. J. Hum. Genet..

[5] Hagerman R.J., Hagerman P. (2016). Fragile X-associated tremor/ataxia syndrome—Features, mechanisms and management. Nat. Rev. Neurol..

[6] Jacquemont S., Hagerman R.J., Leehey M., Hall D., Levine R., Brunberg J., Jardini T., Gane L.W., Harris S.W., Herman K. (2004). Penetrance of the Fragile X–Associated Tremor / Ataxia Syndrome in a Premutation Carrier Population. JAMA.

[7] Folstein M.F., Folestein S.E., McHugh P.R. (1975). Mini-mental state: A practical method for grading the cognitive state of patients for the clinician. J. Psychiatr. Res..

[8] Drozdick L.W., Wahlstrom D., Zhu J., Weiss L.G., Flanagan D., Harrison P. (2012). The Wechsler Adult Intelligence Scale—Fourth Edition and the Wechsler Memory Scale-Fourth Edition. Contemporary Intellectual Assessment: Theories, Tests, and Issues.

[9] Grigsby J., Kaye K. (1996). Behavioral Dyscontrol Scale: Manual.

[10] First M., Williams J., Karg R., Spitzer R. (2015). Structured Clinical Interview for DSM-5—Research Version (SCID-5 for DSM-5, Research Version; SCID-5-RV).

[11] Bacalman S., Farzin F., Bourgeois J., Cogswell J., Goodlin-Jones B., Gane L., Grigsby J., Leehey M., Tassone F., Hagerman R. (2006). Psychiatric Phenotype of the Fragile X-Associated Tremor/Ataxia Syndrome (FXTAS) in Males: Newly Described Fronto-Subcortical Dementia. J. Clin. Psychiatry.

[12] Leehey M.A., Berry-Kravis E., Min S.-J., Hall D.A., Rice C.D., Zhang L., Grigsby J., Greco C.M., Reynolds A., Lara R. (2007). Progression of tremor and ataxia in male carriers of the FMR1 premutation. Mov. Disord..

[13] Juncos J.L., Lazarus J.T., Graves-Allen E., Shubeck L., Rusin M., Novak G., Hamilton D., Rohr J., Sherman S.L. (2011). New clinical findings in the fragile X-associated tremor ataxia syndrome (FXTAS). Neurogenetics.

[14] Zhao C., Liu Y., Wang Y., Li H., Zhang B., Yue Y., Zhang J. (2020). A Chinese case of fragile X-associated tremor/ataxia syndrome (FXTAS) with orthostatic tremor:case report and literature review on tremor in FXTAS. BMC Neurol..

[15] Leehey M.A., Hall D.A., Liu Y., Hagerman R.J., Tassone F., Hall D.A. (2016). Clinical Neurological Phenotype of FXTAS. FXTAS, FXPOI, and Other Premutation Disorders.

[16] Tassone F., Adams J., Berry-Kravis E.M., Cohen S.S., Brusco A., Leehey M.A., Li L., Hagerman R.J., Hagerman P.J. (2007). CGG repeat length correlates with age of onset of motor signs of the fragile X-associated tremor/ataxia syndrome (FXTAS). Am. J. Med. Genet. Part B.

[17] Kamm C., Healy D.G., Quinn N.P., Wüllner U., Moller J.C., Schols L., Geser F., Burk K., Børglum A.D., Pellecchia M.T. (2005). The fragile X tremor ataxia syndrome in the differential diagnosis of multiple system atrophy: Data from the EMSA Study Group. Brain.

[18] Berry-Kravis E., Goetz C.G., Leehey M.A., Hagerman R.J., Zhang L., Li L., Nguyen D., Hall D.A., Tartaglia N., Cogswell J. (2007). Neuropathic features in fragile X premutation carriers. Am. J. Med. Genet. Part A.

[19] O’Keefe J.A., Robertson E.E., Ouyang B., Carns D., McAsey A., Liu Y., Swanson M., Bernard B., Berry-Kravis E., Hall D.A. (2018). Cognitive function impacts gait, functional mobility and falls in fragile X-associated tremor/ataxia syndrome. Gait Posture.

[20] Seritan A., Cogswell J., Grigsby J. (2013). Cognitive Dysfunction in FMR1 Premutation Carriers. Curr. Psychiatry Rev..

[21] Brega A., Goodrich G., Bennett R., Hessl D., Engle K., Leehey M., Bounds L., Paulich M., Hagerman R., Hagerman P. (2008). The Primary Cognitive Deficit among Males with Fragile X-Associated Tremor/Ataxia Syndrome (FXTAS) is a Dysexecutive Syndrome. J. Clin. Exp. Neuropsychol..

[22] O’Keefe J.A., Robertson-Dick E.E., Hall D.A., Berry-Kravis E. (2016). Gait and Functional Mobility Deficits in Fragile X-Associated Tremor/Ataxia Syndrome. Cerebellum.

[23] Jacquemont S., Leehey M.A., Hagerman R.J., Beckett L.A., Hagerman P.J. (2006). Size bias of fragile X premutation alleles in late-onset movement disorders. J. Med. Genet..

[24] Leehey M.A., Berry-Kravis E., Goetz C.G., Zhang L., Hall D.A., Li L., Rice C.D., Lara R., Cogswell J., Reynolds A. (2008). FMR1 CGG repeat length predicts motor dysfunction in premutation carriers. Neurology.

[25] Birch R.C., Hocking D.R., Cornish K.M., Menant J.C., Georgiou-Karistianis N., Godler D.E., Wen W., Hackett A., Rogers C., Trollor J.N. (2015). Preliminary evidence of an effect of cerebellar volume on postural sway in FMR1 premutation males. Genes Brain Behav..

[26] Seaquist E.R. (2010). The Final Frontier: How Does Diabetes Affect the Brain?. Diabetes.

[27] Hajjar I., Quach L., Yang F., Chaves P.H., Newman A.B., Mukamal K., Longstreth W., Inzitari M., Lipsitz L.A. (2011). Hypertension, White Matter Hyperintensities, and Concurrent Impairments in Mobility, Cognition, and Mood. Circulation.

[28] Kim H., Yun C.-H., Thomas R.J., Lee S.H., Seo H.S., Cho E.R., Lee S.K., Yoon D.W., Suh S., Shin C. (2013). Obstructive Sleep Apnea as a Risk Factor for Cerebral White Matter Change in a Middle-Aged and Older General Population. Sleep.

[29] Sachdev P.S. (2005). Homocysteine and brain atrophy. Prog. Neuro-Psychopharmacol. Biol. Psychiatry.

[30] Ligsay A., El-Deeb M., Salcedo-Arellano M.J., Schloemerkemper N., Grayson J.S., Hagerman R. (2019). General Anesthetic Use in Fragile X Spectrum Disorders. J. Neurosurg. Anesthesiol..

[31] Muzar Z., Adams P.E., Schneider A., Hagerman R.J., Lozano R. (2014). Addictive substances may induce a rapid neurological deterioration in fragile X-associated tremor ataxia syndrome: A report of two cases. Intractable Rare Dis. Res..

[32] Hall D.A., Birch R.C., Anheim M., Jønch A.E., Pintado E., O’Keefe J., Trollor J.N., Stebbins G.T., Hagerman R.J., Fahn S. (2014). Emerging topics in FXTAS. J. Neurodev. Disord..

[33] Famula J.L., McKenzie F., McLennan Y.A., Grigsby J., Tassone F., Hessl D., Rivera S.M., Martinez-Cerdeno V., Hagerman R.J. (2018). Presence of middle cerebellar peduncle sign in FMR1 premutation carriers without tremor and ataxia. Front. Neurol..

[34] Adams J.S., Adams P.E., Nguyen D., Brunberg J.A., Tassone F., Zhang W., Koldewyn K., Rivera S.M., Grigsby J., Zhang L. (2007). Volumetric brain changes in females with fragile X-associated tremor/ataxia syndrome (FXTAS). Neurology.

[35] Renaud M., Perriard J., Coudray S., Sévin-Allouet M., Marcel C., Meissner W.G., Chanson J.-B., Collongues N., Philippi N., Gebus O. (2015). Relevance of corpus callosum splenium versus middle cerebellar peduncle hyperintensity for FXTAS diagnosis in clinical practice. J. Neurol..

[36] Hermanson M., Jhaveri M., Stebbins G., Dunn E., Merkitch D., Berry-Kravis E., Hall D. (2015). The Splenium of the Corpus Callosum Sign in Fragile X associated Tremor Ataxia Syndrome (FXTAS) (P2.125). Neurology.

[37] Hall D.A., Robertson E., Shelton A.L., Losh M.C., Mila M., Moreno E.G., Gomez-Anson B., Martínez-Cerdeño V., Grigsby J., Lozano R. (2016). Update on the Clinical, Radiographic, and Neurobehavioral Manifestations in FXTAS and FMR1 Premutation Carriers. Cerebellum.

[38] Wang J.Y., Hagerman R.J., Rivera S.M. (2013). A multimodal imaging analysis of subcortical gray matter in fragile X premutation carriers. Mov. Disord..

[39] Hashimoto R., Javan A.K., Tassone F., Hagerman R.J., Rivera S.M. (2011). A voxel-based morphometry study of grey matter loss in fragile X-associated tremor/ataxia syndrome. Brain.

[40] Cohen S., Masyn K., Adams J., Hessl D., Rivera S., Tassone F., Brunberg J., DeCarli C., Zhang L., Cogswell J. (2006). Molecular and imaging correlates of the fragile X–associated tremor/ataxia syndrome. Neurology.

[41] Brunberg J.A., Jacquemont S., Hagerman R.J., Berry-Kravis E.M., Grigsby J., Leehey M.A., Tassone F., Brown W.T., Greco C.M., Hagerman P.J. (2002). Fragile X Premutation Carriers: Characteristic MR Imaging Findings of Adult Male Patients with Progressive Cerebellar and Cognitive Dysfunction. Am. J. Neuroradiol..

[42] Ariza J., Steward C., Rueckert F., Widdison M., Coffman R., Afjei A., Noctor S.C., Hagerman R., Hagerman P., Martínez-Cerdeño V. (2015). Dysregulated iron metabolism in the choroid plexus in fragile X-associated tremor/ataxia syndrome. Brain Res..

[43] Ariza J., Rogers H., Hartvigsen A., Snell M., Dill M., Judd D., Hagerman P., Martínez-Cerdeño V. (2017). Iron accumulation and dysregulation in the putamen in fragile X-associated tremor/ataxia syndrome. Mov. Disord..

[44] Wang J.Y., Hessl D., Iwahashi C., Cheung K., Schneider A., Hagerman R.J., Hagerman P.J., Rivera S.M. (2013). Influence of the fragile X mental retardation (FMR1) gene on the brain and working memory in men with normal FMR1 alleles. Neuroimage.

[45] Hashimoto R., Srivastava S., Tassone F., Hagerman R.J., Rivera S.M. (2011). Diffusion tensor imaging in male premutation carriers of the fragile X mental retardation gene. Mov. Disord..

